# Supplementary material for: Targeting PRMT3 impairs methylation and oligomerization of HSP60 to boost anti-tumor immunity by activating cGAS/STING signaling
Source: Nat Commun. 2024 Sep 10;15:7930. doi: 10.1038/s41467-024-52170-3 (PMC11387718; doi:10.1038/s41467-024-52170-3)
Supplement: Supplementary file 1 — Supplementary Information [file 41467_2024_52170_MOESM1_ESM.pdf]

Supplementary information

Targeting PRMT3 Impairs Methylation and Oligomerization of HSP60 to Boost Anti-Tumor Immunity by Activating cGAS/STING Signaling

Yunxing Shi[1,2,3#], Zongfeng Wu[1,2#], Shaoru Liu[1,2#], Dinglan Zuo[1,2#], Yi Niu[1,2], Yuxiong Qiu[1,2], Liang Qiao[1,2], Wei He[1,2], Jiliang Qiu[1,2], Yunfei Yuan[1,2*], Guocan Wang[4*], Binkui Li[1,2*]

[1]State Key Laboratory of Oncology in South China, Guangdong Provincial Clinical Research for Cancer, Sun Yat-Sen University Cancer Center, Guangzhou, China.

[2]Department of Liver Surgery, Sun Yat-Sen University Cancer Center, Guangzhou, China.

[3]Department of Colorectal Surgery, Guangdong Institute of Gastroenterology, and Guangdong Provincial Key Laboratory of Colorectal and Pelvic Floor Diseases, The Sixth Affiliated Hospital of Sun Yat-sen University, Guangzhou, China

Supplementary fig 2-16 and figure legend--2-25

Supplementary Table 1. Antibodies included in the study-------------------------------------26

Supplementary Table 2. The primers used for qPCR assay in present study-----------27

Supplementary Table 3. Sequences of RNA Oligonucleotides-------------------------------28

Supplementary Table 4. The primers used for CHIP assay in present study------------29

Supplementary Figures

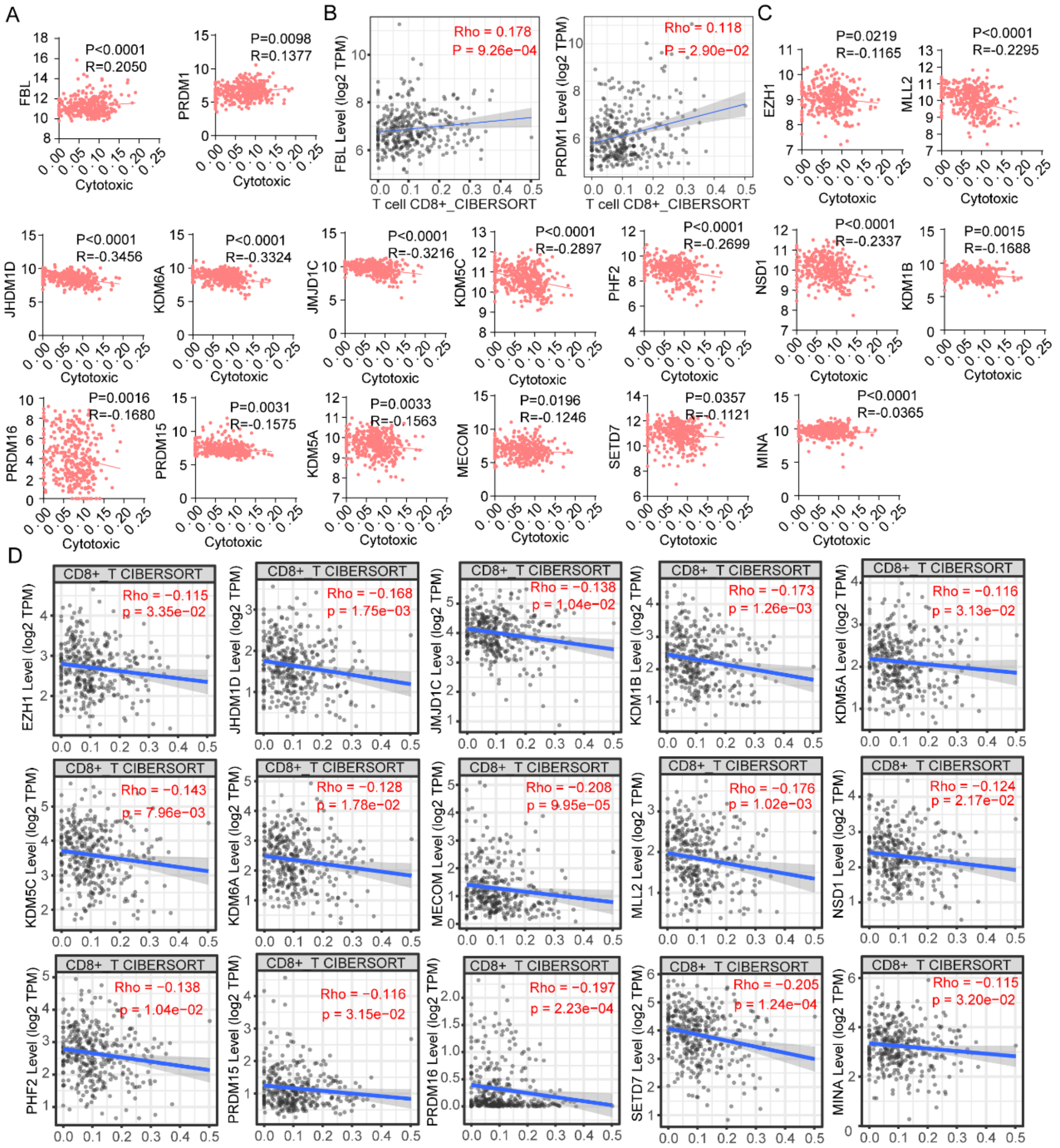

Supplementary Figure 1. High PRMT3 expression was strongly associated with poorer response to immunotherapy in HCC patients

A. Positive correlation between PTM expression and cytotoxic T cells score evaluated by ImmuneCell AI in TCGA-LIHC dataset.

B. Positive correlation between PTM expression and CD8+ T cell infiltration evaluated by CIBERSORT in TCGA-LIHC dataset.

C. Negative correlation between PTM expression and cytotoxic T cells score evaluated by ImmuneCell AI in TCGA-LIHC dataset.

D. Negative correlation between PTM expression and CD8+ T cell infiltration evaluated by CIBERSORT in TCGA-LIHC dataset.

Source data are provided as a Source Data file.

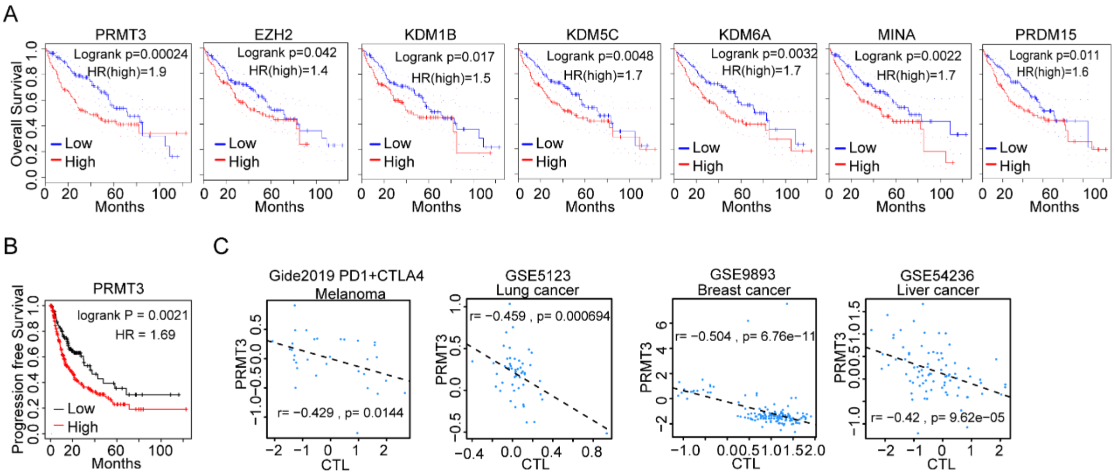

Supplementary Figure 2. High PRMT3 expression was strongly associated with poorer response to immunotherapy in HCC patients

A. Kaplan–Meier overall survival curves of individuals with different PTM expression in TCGA-LIHC dataset.

B. Kaplan–Meier progression-free survival curves of individuals with different PRMT3 expression in TCGA-LIHC dataset.

C. PRMT3 was negatively correlated with immune infiltration in several public dataset.

Source data are provided as a Source Data file.

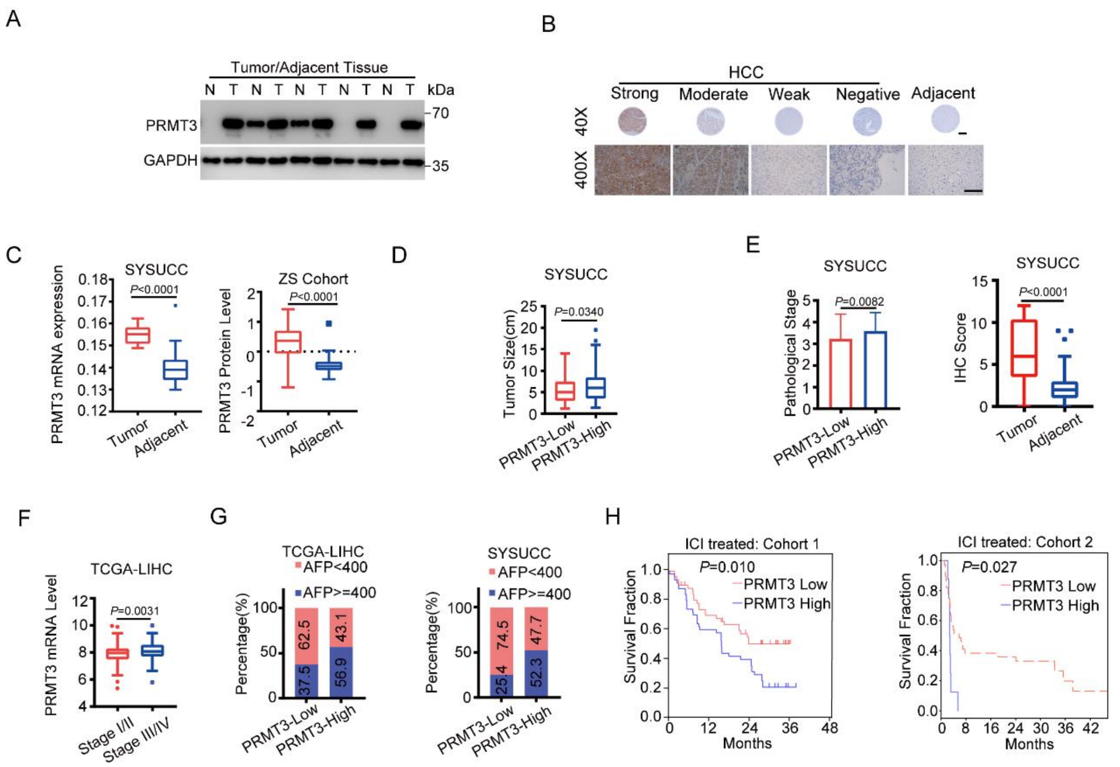

Supplementary Figure 3. PRMT3 was up-regulated in HCC and predicts a poor prognosis

A. PRMT3 expression was detected by western blot in HCC and adjacent tissue (n=3 biologically independent samples).

B. PRMT3 expression was detected by IHC in HCC and adjacent tissue.

C. PRMT3 expression was up-regulated in the SYSUCC cohort and a public dataset.

D. PRMT3 expression was detected by IHC in different sizes of tumors.

E. PRMT3 expression was detected by IHC in HCC of different pathological stages.

F. PRMT3 expression was detected by IHC in HCC of different TNM stages.

G. The correlation between PRMT3 and AFP expression levels.

H. Associations of PRMT3 expression with overall survival in public immunotherapy datasets.

Data in **C**, **D**, **E** and **F** are presented as mean ± SD. Data were analyzed by two-sided Student's t test in **C**, **D**, **E** and **F**. Source data are provided as a Source Data file.

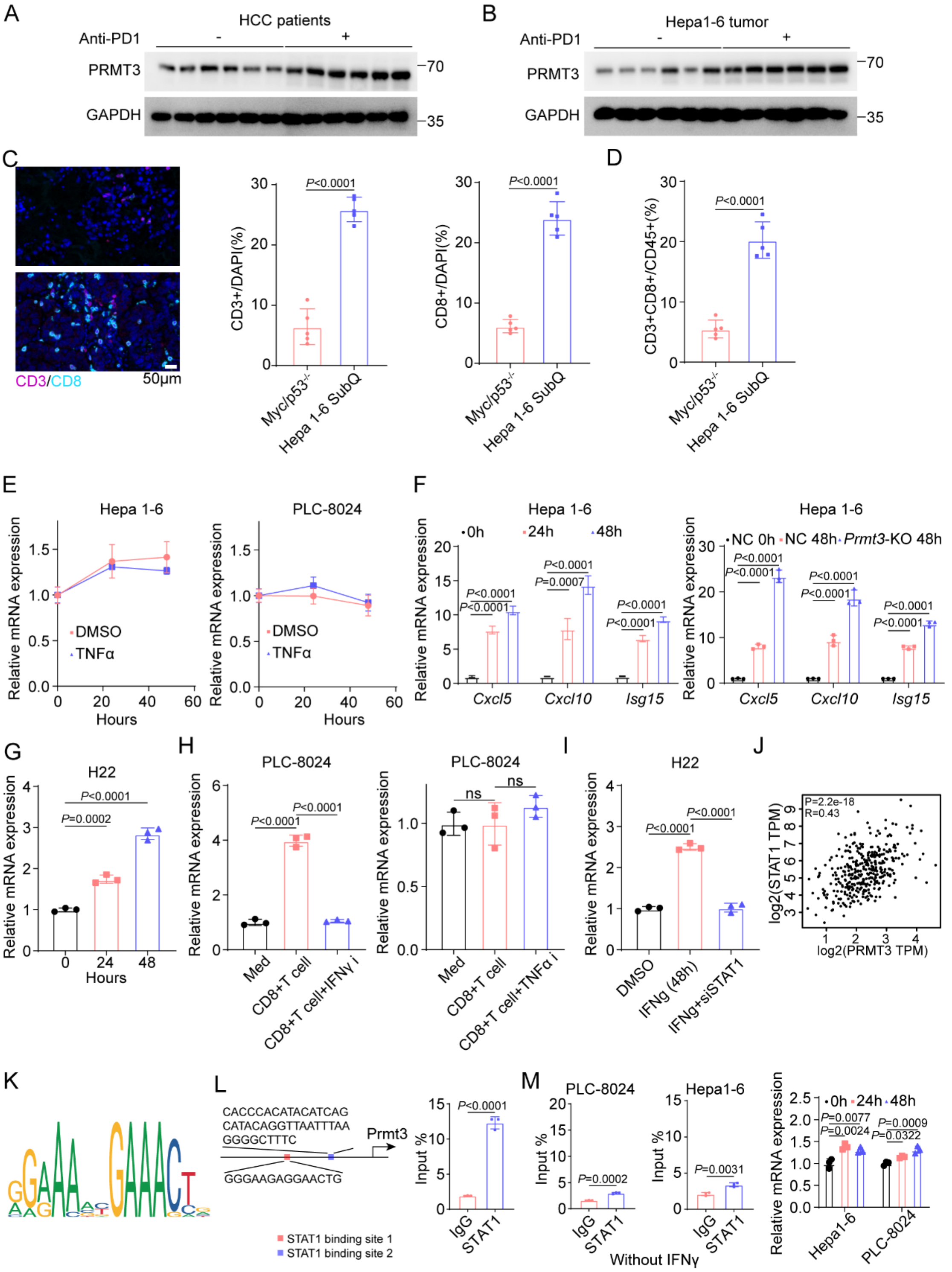

Supplementary Figure 4. Effector T cells up-regulate PRMT3 expression via IFN-γ-STAT1-dependent pathways

A. PRMT3 expression was detected by western blot in HCC patients who received anti-PD1 therapy or not (n=3 biologically independent samples).

B. PRMT3 expression was detected by western blot and qPCR in Hepa1-6

subcutaneous tumors which received anti-PD1 therapy or not (n=3 biologically independent samples).

C. Immunofluorescence identifying CD3+ and CD8+ immune cells in subcutaneous tumors and Myc/TP53$^{-/-}$ spontaneous tumors (n=5). Scale bar, 50 μm.

D. Flow cytometry analysis assessing the percentage of CD8+ T cell from tumors in indicated groups (n=5). Data was analyzed by FlowJo.

E. Hepa1-6 and PLC-8024 cells were treated with TNFα. PRMT3 expression was analyzed by qPCR (n=3 biologically independent samples).

F. Several interferon-stimulated genes were detected by qPCR in Hepa1-6 cells treated with IFNγ (n=3 biologically independent samples).

G. H22 cells were treated with IFNγ. PRMT3 expression was analyzed by qPCR (n=3 biologically independent samples).

H. PLC-8024 cells were treated with the conditioned medium of CD8^{+} T cells sorted from HCC tumors and inhibitors of IFNγ and TNFα. PRMT3 expression was analyzed by qPCR (n=3 biologically independent samples).

I. PRMT3 expression in *Stat1*-KD and control H22 cells as shown by qRT-PCR (n=3 biologically independent samples).

J. Correlation analysis between STAT1 and PRMT3 expression in TCGA-LIHC dataset.

K. The binding motif of STAT1 predicted by JASPAR.

L. ChIP-qPCR was used to determine the binding of STAT1 to *Prmt3* promoter region in Hepa1-6 cells treated with IFNγ (n=3 biologically independent samples).

M. Several interferon-stimulated genes were detected by qPCR in Hepa1-6 cells treated with IFNα (n=3 biologically independent samples).

Data in **C-I**, **L** and **M** are presented as mean ± SD. Data were analyzed by two-sided Student's t test in **C, D, E and K,** by one way ANOVA in **F-I** and **M**. Source data are provided as a Source Data file.

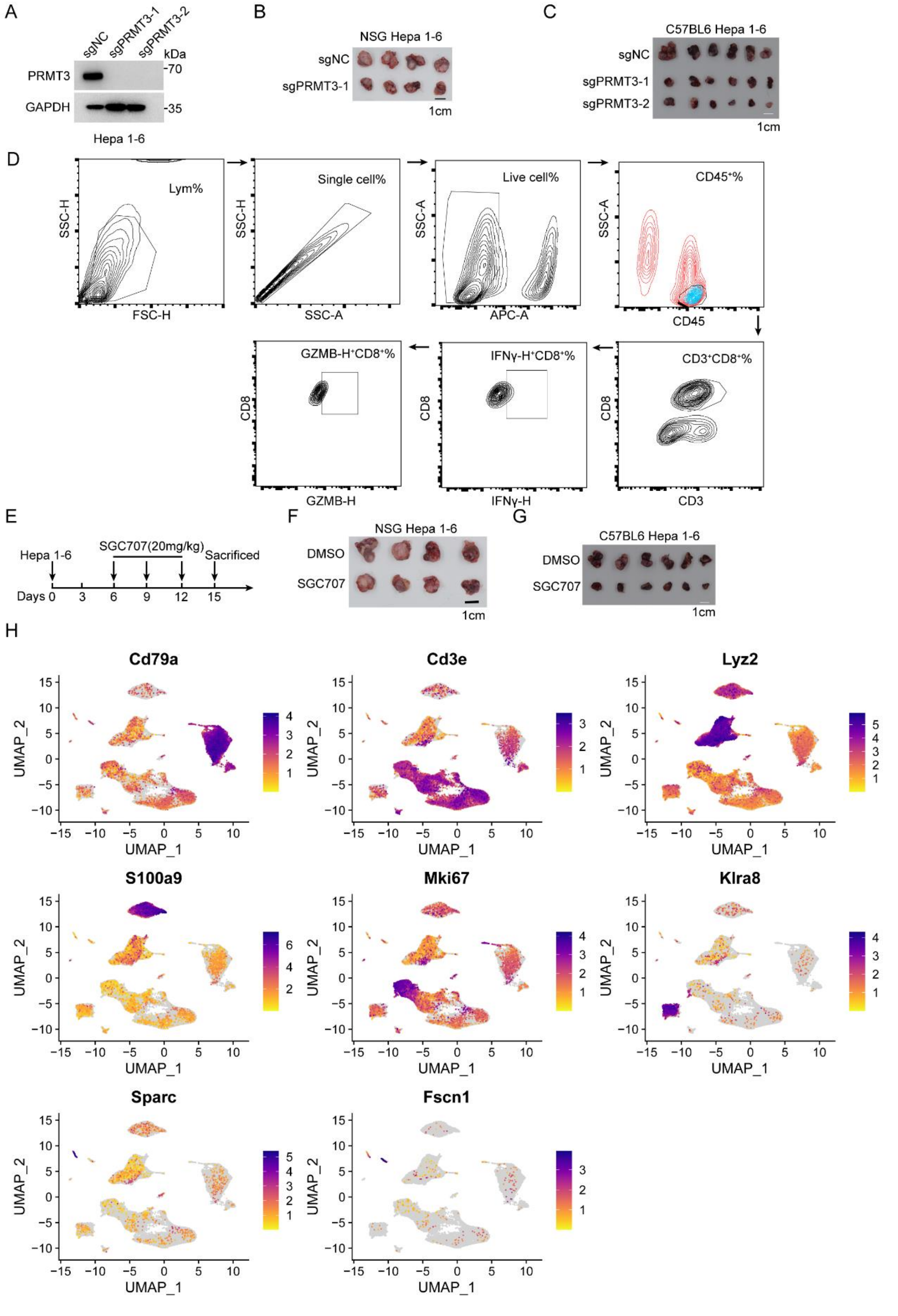

Supplementary Figure 5. *Prmt3*-KO or PRMT3 inhibition increases immune infiltration and activates T cell-mediated anti-tumor immunity

A. PRMT3 expression was detected by western blot in *Prmt3*-KO and ctrl Hepa1-6 cells.

B. The effect of *Prmt3*-KO on subcutaneous tumor growth in NSG mice (n=5).

C. The effect of *Prmt3*-KO on subcutaneous tumor growth in C57BL6 mice (n=6).

D. Gating strategies for flow cytometry. Identification of $CD8^+T$, $IFN\gamma^+$ $CD8^+T$ cells and $GZMB^+$ $CD8^+T$ cells.

E. Schematic diagram illustrates the workflow of Hepa1-6 subcutaneous model treated with DMSO or SGC707 (20 mg/kg) (n=6).

F. The effect of DMSO or SGC707 (20 mg/kg) on subcutaneous tumor growth in NSG mice (n=5).

G. The effect of DMSO or SGC707 (20 mg/kg) on subcutaneous tumor growth in C57BL6 mice (n=6).

H. Feature plot for clustering analysis in scRNA-seq.

Source data are provided as a Source Data file.

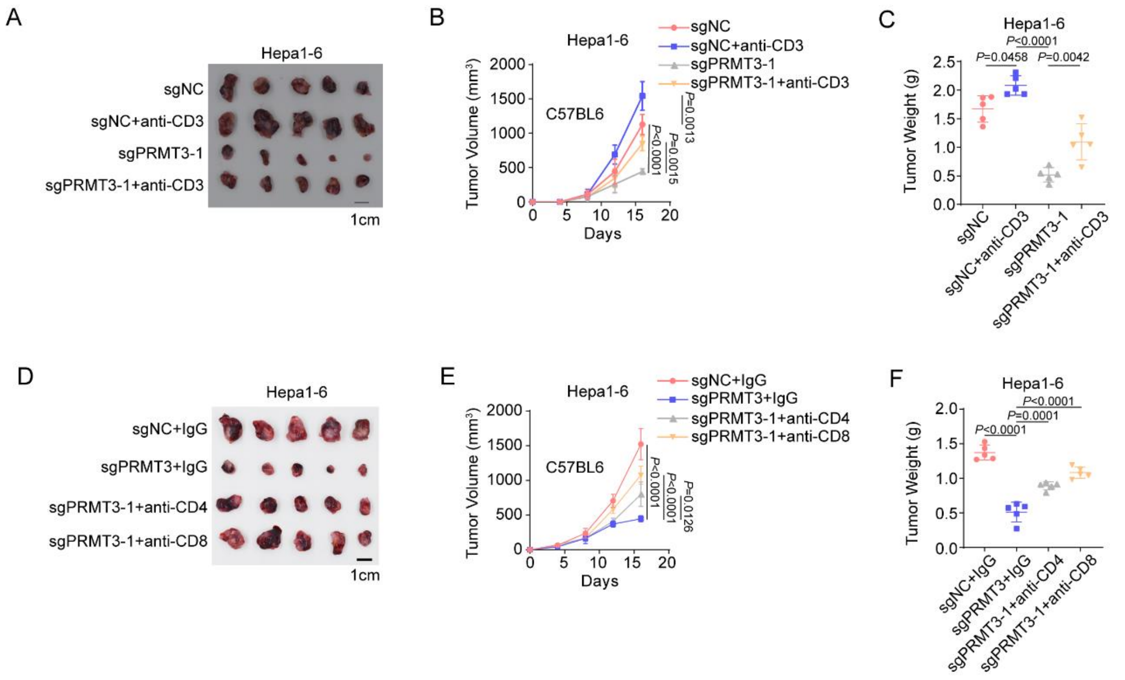

Supplementary Figure 6. T cells mediated the effect of PRMT3 on anti-tumor immunity in HCC

A. The effect of *Prmt3*-KO and anti-CD3 antibody treatment on subcutaneous tumor growth in C57BL6 mice (n=5).

B, C. The measurement of tumor volumes (B) and tumor weights (C) of subcutaneously implanted Hepa1-6 cells (*Prmt3*-KO and *Prmt3*-WT) treated with anti-CD3 antibody in C57BL6 mice (n=5).

D. The effect of *Prmt3*-KO and anti-CD4/CD8 antibody treatment on subcutaneous tumor growth in C57BL6 mice (n=5).

E, F. The measurement of tumor volumes (B) and tumor weights (C) of subcutaneously implanted Hepa1-6 cells (*Prmt3*-KO and *Prmt3*-WT) treated with anti-CD4/CD8 antibody in C57BL6 mice (n=5).

Data in **B**, **C**, **E** and **F** are presented as mean ± SD. Data were analyzed by one way

ANOVA in **B**, **C**, **E** and **F**. Source data are provided as a Source Data file.

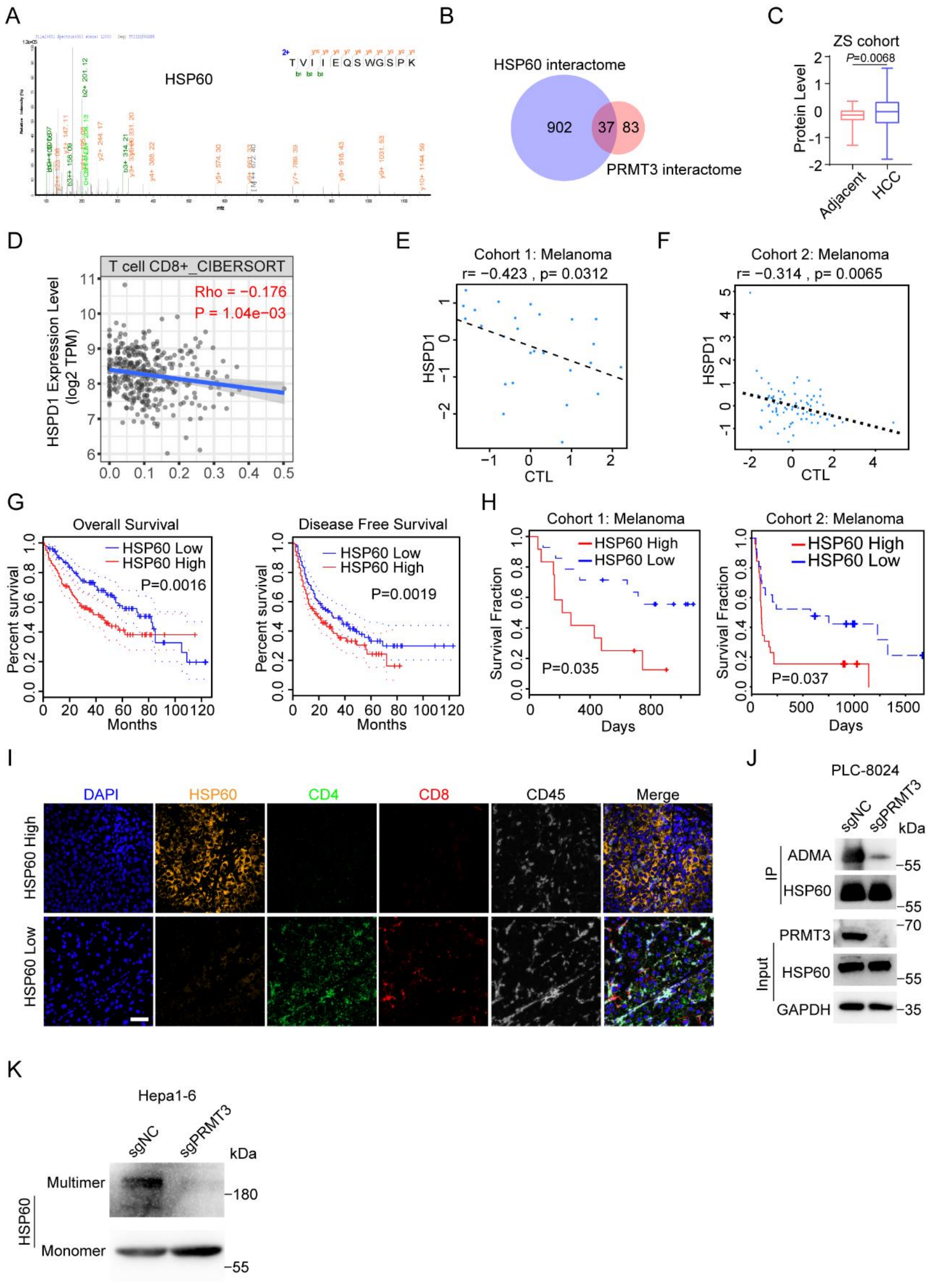

Supplementary Figure 7. PRMT3 methylates HSP60 at R446 and promotes its oligomerization

A. Fragmentation spectrum of the HSP60 peptide identified by liquid chromatography/tandem mass spectrometry (LC-MS/MS).

B. Venn Diagram showing the 37(1/3) proteins were identified for both PRMT3 and HSP60 interacting proteins retrieved from the BioRID database.

C. HSP60 was significantly up-regulated in HCC compared to adjacent tissue in a public proteomics dataset.

D. Associations of HSPD1 expression with $CD8^+$ T cell infiltration evaluated by CIBERSORT in TCGA-LIHC dataset.

E, F. Associations of *HSPD1* expression with CTL infiltration in two immunotherapy cohorts.

G. Kaplan–Meier overall survival curves of individuals with different *HSPD1* expression in TCGA-LIHC dataset.

H. Kaplan–Meier overall survival curves of individuals with different *HSPD1* expression in two immunotherapy cohorts.

I. Representative example of HSP60-high and HSP60-low HCC. Tumor staining by multiplexed IHC shows the spatial distributions of $CD4^+$ T cells and $CD8^+$ T cells. Scale bar: 50μm.

J. WB analysis of immunoprecipitated HSP60 to determine the effect of *Prmt3*-KO on arginine methylation of HSP60 in Hepa1-6 cells using asymmetric dimethylarginine antibody (ADMA) (n=3 biologically independent samples).

K. WB analysis determined the effect of PRMT3-KO on the oligomerization of HSP60 in Hepa1-6 cells (n=3 biologically independent samples).

Data in **C** is presented as mean ± SD. Data was analyzed by two-sided Student's t test in **C**. Source data are provided as a Source Data file.

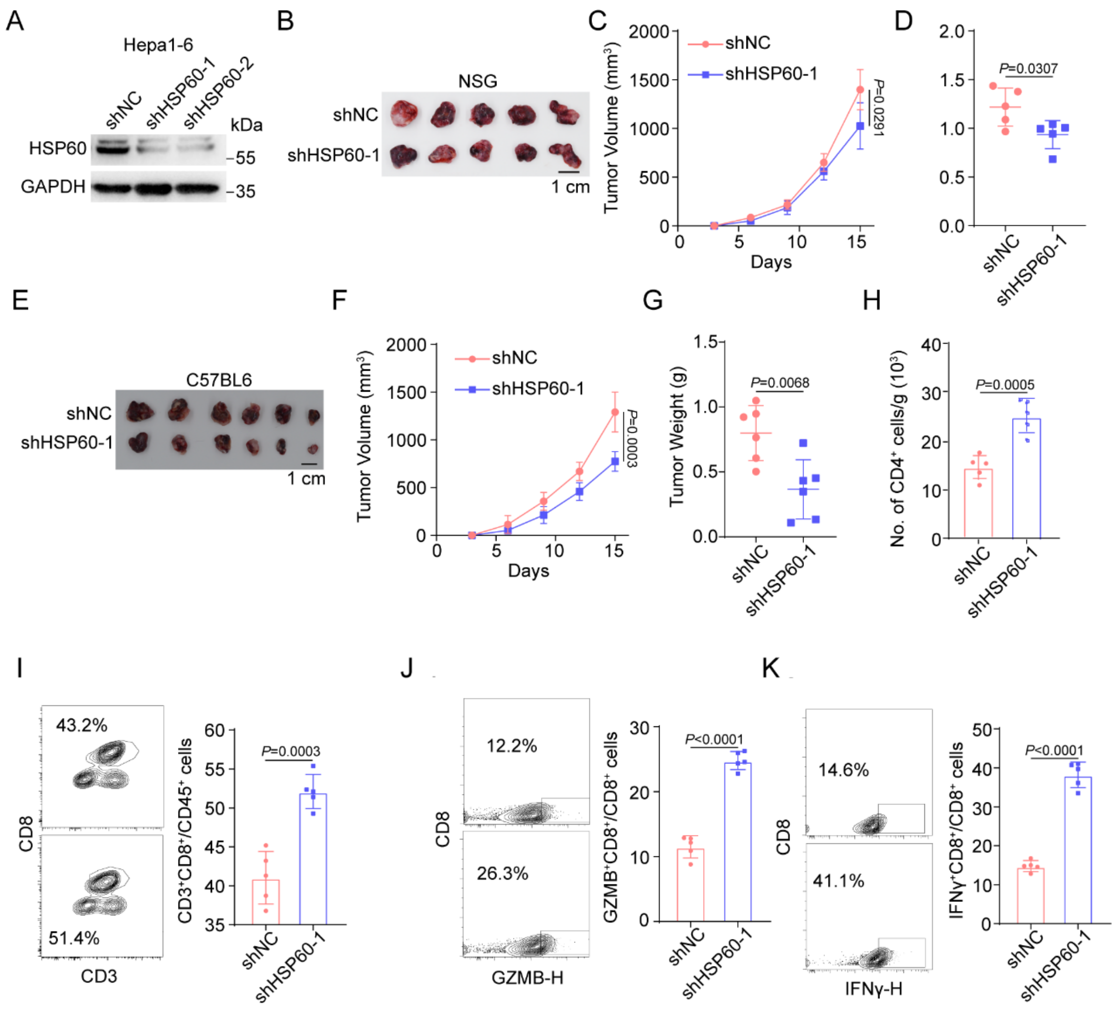

Supplementary Figure 8. HSP60 KD inhibited tumor growth and activated anti-tumor immunity

A. HSP60 expression was detected by western blot in HSP60-KD and ctrl Hepa1-6 cells (n=3 biologically independent samples).

B. The effect of HSP60-KD on subcutaneous tumor growth in NSG mice (n=5).

C, D. The measurement of tumor volumes (C) and tumor weights (D) of subcutaneously implanted indicated Hepa1-6 cells in NSG mice (n=5).

E. The effect of HSP60-KD on subcutaneous tumor growth in C57BL6 mice (n=6).

F, G. The measurement of tumor volumes (F) and tumor weights (G) of subcutaneously implanted indicated Hepa1-6 cells in C57BL6 mice (n=6).

H, I. $CD4^+$ (H) and $CD8^+$ (I) T cells from tumors in ctrl and HSP60-KD groups were analyzed by flow cytometry (n=5).

J, K. Flow cytometry analysis assessing the percentage of T cell functional markers IFNγ (J) and GZMB (K) from tumors in ctrl and HSP60-KD groups (n=5).

Data in **C**, **D** and **F-K** are presented as mean ± SD. Data were analyzed by two-sided Student's t test in **C**, **D** and **F-K**. Source data are provided as a Source Data file.

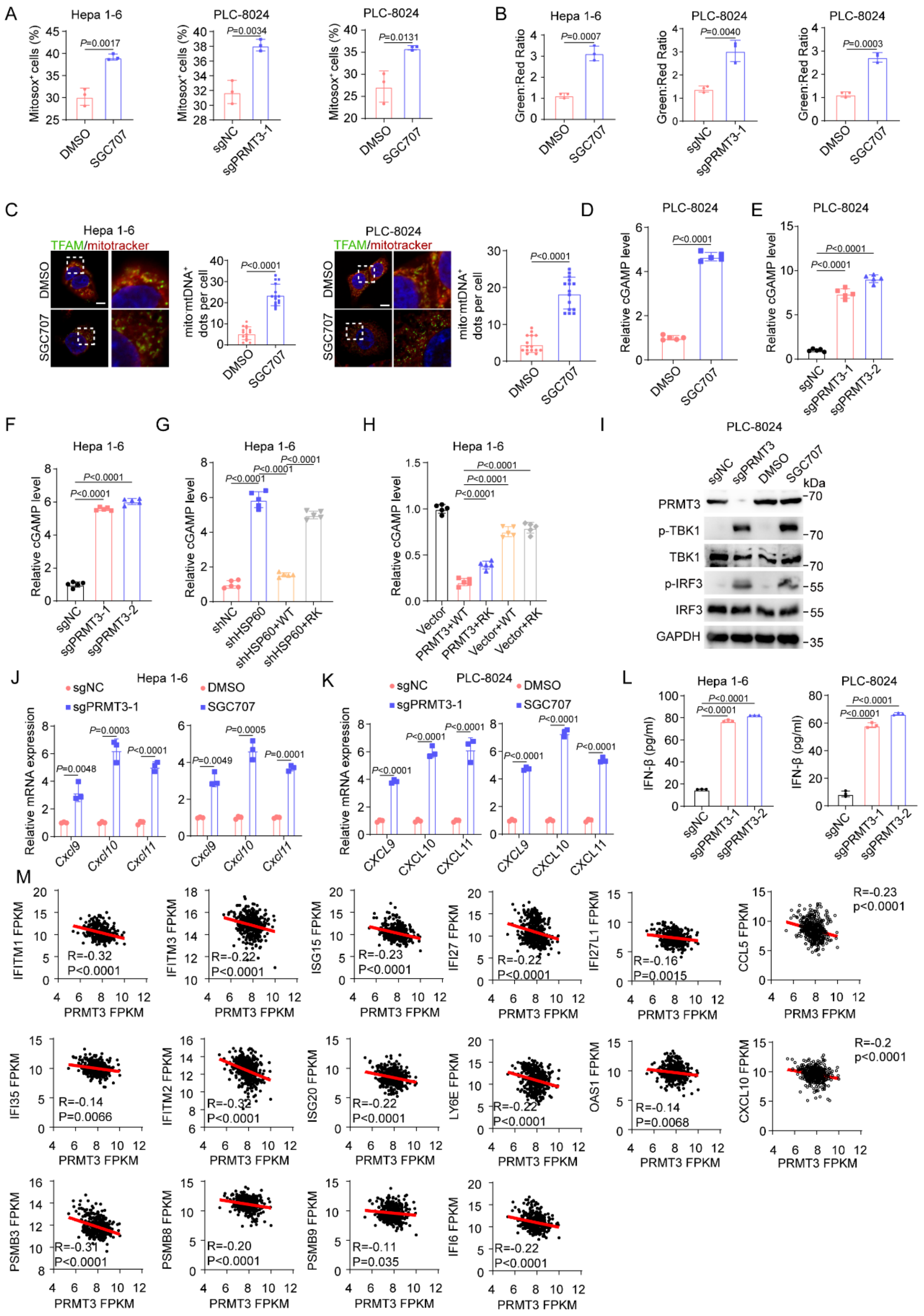

Supplementary Figure 9. Inhibition of PRMT3-mediated arginine methylation of HSP60 induces mtDNA leakage and cGAS/STING signaling activation

A. Mitosox+ cells were detected by flow cytometry in indicated groups (n=3 biologically independent samples).

B. JC-1 aggregates: JC-1 monomers ratio in indicated groups (n=3 biologically independent samples).

C. The effect of SGC707 treatment on mtDNA release was assessed by IF staining with mitotracker and TFAM. Representative images (scale bar, 5 μm) and quantitative results are shown (n=15).

D-H. cGAMP level was analyzed in indicated groups with ELISA assay (n=5 biologically independent samples).

I. The effects of *PRMT3*-KO or SGC707 treatment on expression of TBK1/p-TBK1 and IRF3/p-IRF3 in PLC-8024 cells (n=3 biologically independent samples).

J. The effects of *PRMT3*-KO and SGC707 treatment on expression of interferon-stimulated genes in Hepa1-6 cells (n=3 biologically independent samples).

K. The effects of *PRMT3*-KO and SGC707 treatment on expression of interferon-stimulated genes in PLC-8024 cells (n=3 biologically independent samples).

L. The effects of *PRMT3*-KO on IFNβ production in Hepa1-6 and PLC-8024 cells (n=3 biologically independent samples).

M. Correlation between several interferon-stimulated genes and *PRMT3* in TCGA-LIHC dataset.

Data in **A-H** and **J-L** are presented as mean ± SD. Data were analyzed by two-sided Student's t test in **A-H** and **J-L,** by one way ANOVA in **E-H**, **L**. Source data are provided as a Source Data file.

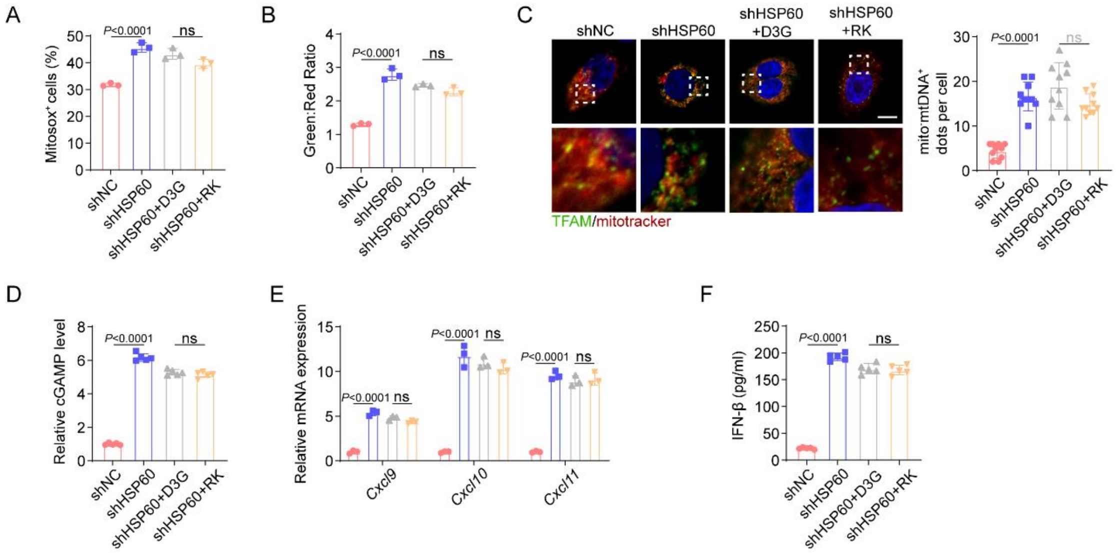

Supplementary Figure 10. Both HSP60-D3G and HSP60-RK mutant induces mtDNA leakage and cGAS/STING signaling activation

A. Mitosox+ cells were detected by flow cytometry in indicated groups (n=3 biologically independent samples).

B. JC-1 aggregates: JC-1 monomers ratio in indicated groups (n=3 biologically independent samples).

C. The effect of SGC707 treatment on mtDNA release was assessed by IF staining with mitotracker and TFAM. Representative images (scale bar, 5 μm) and quantitative results are shown (n=15).

D. cGAMP level was analyzed in indicated groups with ELISA assay (n=5 biologically independent samples).

E. The effects of HSP60-D3G and HSP60-RK mutant on expression of interferon-stimulated genes in Hepa1-6 cells (n=5 biologically independent samples).

F. The effects of HSP60-D3G and HSP60-RK mutant on IFNβ production in Hepa1-6 (n=3 biologically independent samples).

Data in **A-F** are presented as mean ± SD. Data were analyzed by one way ANOVA in **A-F**. Source data are provided as a Source Data file.

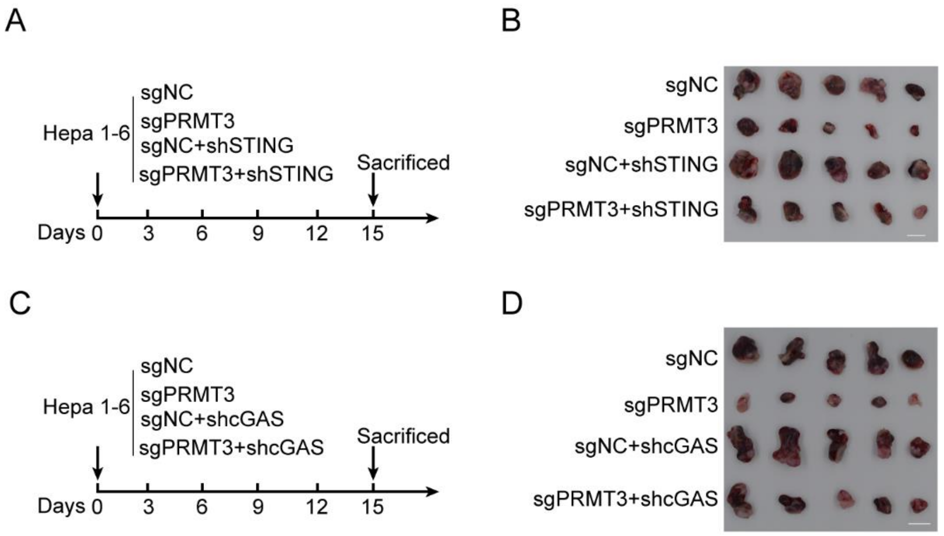

Supplementary Figure 11. The anti-tumor immunity induced by *Prmt3*-KO or inhibition was dependent on the activation of cGAS/STING signaling

A. Schematic diagram illustrates the workflow of subcutaneous model implanted indicated Hepa1-6 cells (n=5).

B. The effect of STING-KD on the tumor growth of subcutaneously implanted *Prmt3*-KO Hepa1-6 cells (n=5). Scale bars, 1 cm.

C. Schematic diagram illustrates the workflow of subcutaneous model implanted indicated Hepa1-6 cells (n=5).

D. The effect of cGAS-KD on the tumor growth of subcutaneously implanted *Prmt3*-KO Hepa1-6 cells (n=5). Scale bars, 1 cm.

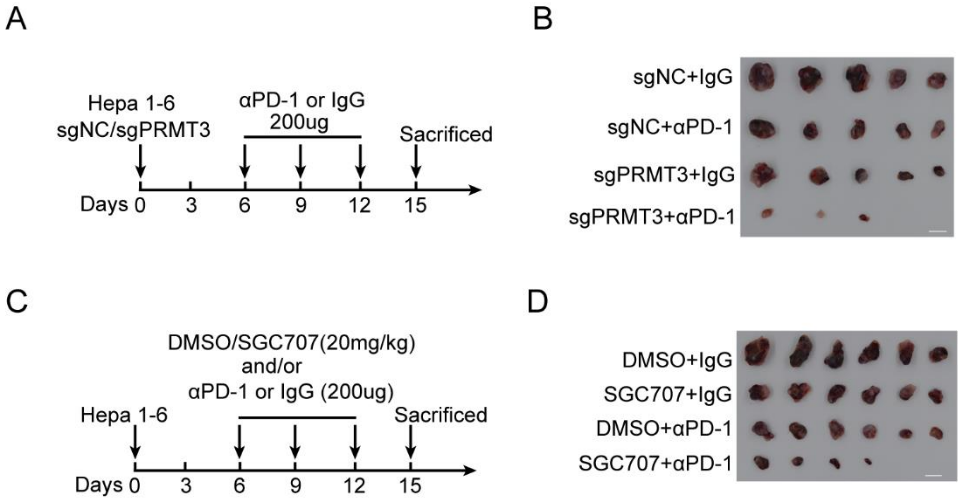

Supplementary Figure 12. *Prmt3*-KO or inhibition enhances immunotherapy response in HCC

A. Schematic diagram illustrates the workflow of subcutaneous model implanted indicated Hepa1-6 cells and treated with PD-1 antibody or IgG (n=5).

B. The effect of *Prmt3*-KO on the tumor growth of subcutaneous tumor growth treated with PD-1 antibody or IgG (n=5). Scale bars, 1 cm.

C. Schematic diagram illustrates the workflow of the subcutaneous model treated with SGC707 and/or PD-1 antibody (n=6).

D. The effect of SGC707 treatment on the tumor growth of subcutaneous tumors treated with PD-1 antibody or IgG (n=5). Scale bars, 1 cm.

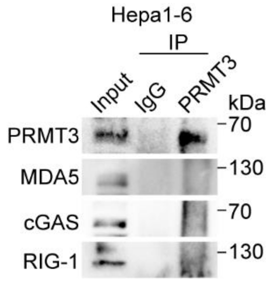

Supplementary Figure 13. WB analysis showed that endogenous PRMT3 did not interact with MDA5, cGAS and RIG-1 in Hepa1-6 cells using reciprocal co-immunoprecipitation (n=3 biologically independent samples).

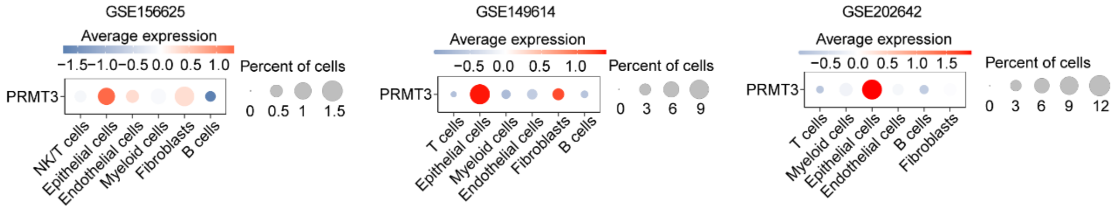

Supplementary Figure 14. PRMT3 expression in various cell types in the tumor niche was analyzed in several public dataset.

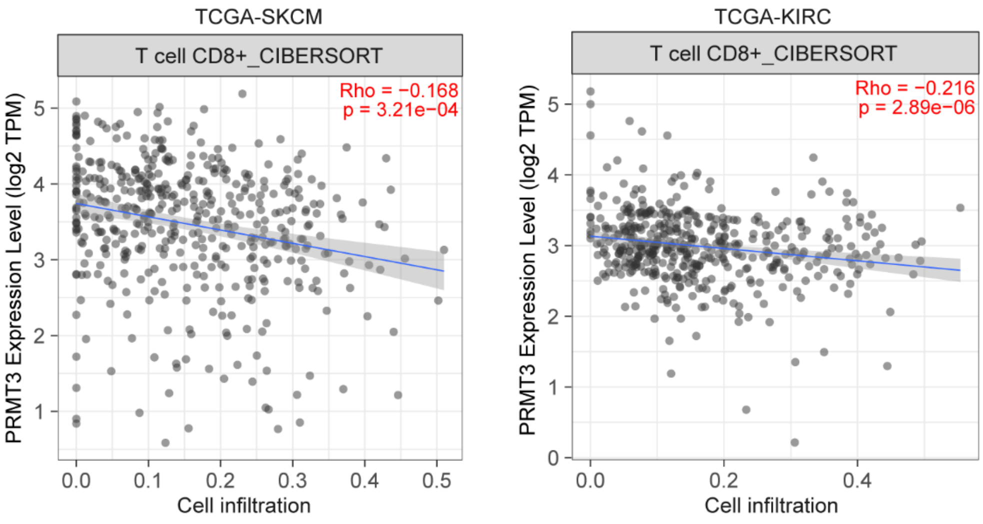

Supplementary Figure 15. Associations of PRMT3 expression with CD8+ T cell infiltration evaluated by CIBERSORT in TCGA-SKCM and TCGA-KIRC dataset.

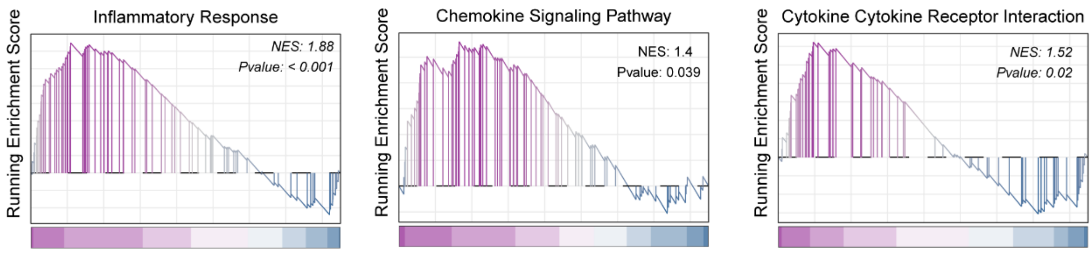

Supplementary Figure 16. Gene set enrichment analysis identified inflammation-related pathways in PRMT3 -KD cells.

Supplementary Table 1. Antibodies included in the study

Antibodies	IDENTIFIER	Catalogue No.	Host species	Species	Application and dilution
Anti-GAPDH	Proteintech	60004-1-Ig	Mouse	All	WB (1:2000)
Anti-PRMT3	Abcam	Ab191562	Rabbit	Hu	WB (1:2000), IHC (1:100), IF (1:100), IP (1:50)
Anti-PRMT3	Proteintech	17628-1-AP	Rabbit	All	WB (1:1000), IP (4ug)
Anti-FLAG	Cell Signaling Technology	#14793	Rabbit	All	WB (1:1000), IP (4ug)
Anti-STAT1	Proteintech	10144-2-AP	Rabbit	All	WB (1:750), CHIP (1:50)
Anti-HSP60	Proteintech	15282-1-AP	Rabbit	All	WB (1:1000), IP (4ug), IF (1:500)
Anti-HSP60	Proteintech	66041-1-Ig	Mouse	All	WB (1:1000), IP (4ug), IF (1:500)
Anti-ADMA	Cell Signaling Technology	13522S	Rabbit	All	WB (1:1000)
Anti-TFAM	Proteintech	CL488-22586	Rabbit	All	IF (1:200)
Anti-dsDNA	Sigma-Aldrich	Cbl186	Mouse	All	IF (1:200)
Anti-TBK1	Proteintech	28397-1-AP	Rabbit	All	WB (1:1000)
Anti-p-TBK1	Cell Signaling Technology	5483S	Rabbit	Hu/Mo	WB (1:1000)
Anti-IRF3	Proteintech	11312-1-AP	Rabbit	All	WB (1:1000)
Anti-p-IRF3	Cell Signaling Technology	29047S#	Rabbit	All	WB (1:1000)
Anti-STING	Proteintech	19851-1-AP	Rabbit	All	WB (1:1000)
Anti-p-STING	Cell Signaling Technology	# 19781S	Rabbit	All	WB (1:1000)
Alexa Fluor 488	Thermo Fisher Scientific	A-11001	Goat	Mo	IF (1:200)
Alexa Fluor 594	Thermo Fisher Scientific	# R37117	Goat	Rat	IF (1:200)
Anti-mouse IgG	Cell Signaling Technology	7076S	Hr	Mo	WB (1:3000)
Anti-rabbit IgG	Cell Signaling Technology	7074S	Hr	Mo	WB (1:3000)
HRP Rabbit/Mouse	DAKO	K5007	NA	Rat, Mo	IHC (NA)
Rabbit IgG	Proteintech	B900610	Rabbit	All	IP (2ug)
Anti-Mouse CD45	BD Pharmingen	557659	Rat	Mouse	Flow cytometry (2.5ul)
Anti-Mouse CD3e	BD Horizon	563565	Hamster	Mouse	Flow cytometry (2.5ul)
Anti-Granzyme B	Invitrogen	17-8898-82	Rat	Mouse	Flow cytometry (2.5ul)
Anti-Mouse IFN-γ	BD Horizon	563376	Rat	Mouse	Flow cytometry (2.5ul)
Anti-Mouse CD8a	BD Pharmingen	553030	Rat	Mouse	Flow cytometry (2.5ul)

Supplementary Table 2. The primers used for qPCR assay in present study

Primer names	Forward (5'-3')	Reverse (5'-3')
PRMT3	CACTGTCTGCTGAAGCCGCATT	GTAGATGACGAGCAGGTTCTGAC
Prmt3	CGACCAGTCTGAAATCCTCTACC	CCACCTTTTCCACAGGAAGACTC
Ccl5	CCTGCTGCTTTGCCTACCTCTC	ACACACTTGGCGGTTCCTTCGA
Cxcl10	ATCATCCCTGCGAGCCTATCCT	GACCTTTTTTGGCTAAACGCTTTC
Cxcl9	CCTAGTGATAAGGAATGCACGATG	CTAGGCAGGTTTGATCTCCGTTC
Cxcl11	CCGAGTAACGGCTGCGACAAAG	CCTGCATTATGAGGCGAGCTTG
ISG15	CTCTGAGCATCCTGGTGAGGAA	AAGGTCAGCCAGAACAGGTCGT
Isg15	CATCCTGGTGAGGAACGAAAGG	CTCAGCCAGAACTGGTCTTCGT
IFITM1	GGCTTCATAGCATTCGCCTACTC	AGATGTTCAGGCACTTGGCGGT
Ifitm1	GCCACCACAATCAACATGCCTG	ACCCACCATCTTCCTGTCCCTA
IFITM3	CTGGGCTTCATAGCATTCGCCT	AGATGTTCAGGCACTTGGCGGT
Ifitm3	TTCTGCTGCCTGGGCTTCATAG	ACCAAGGTGCTGATGTTCAGGC
IFNB1	CTTGGATTCCTACAAAGAAGCAGC	TCCTCCTTCTGGAACTGCTGCA
Ifnb1	GCCTTTGCCATCCAAGAGATGC	ACACTGTCTGCTGGTGGAGTTC
Tnfa	GGTGCCTATGTCTCAGCCTCTT	GCCATAGAACTGATGAGAGGGAG
ACTB	CACCATTGGCAATGAGCGGTTC	AGGTCTTTGCGGATGTCCACGT
Actb	CATTGCTGACAGGATGCAGAAGG	TGCTGGAAGGTGGACAGTGAGG

Supplementary Table 3. Sequences of RNA Oligonucleotides

Name	Sense strand/sense primer (5'-3')	Antisense strand/antisense primer (5'-3')
**siRNA**		
siSTAT1	GGUGCAUUAUGGGCUUCAUTT	AUGAAGCCCAUAAUGCACCTT
**shRNA**		
shHSP60-1	GCAGGTTCCTCACCAATAACT	AGTTATTGGTGAGGAACCTGC
shHSP60-2	GACGATATATAATGGTTTACT	AGTAAACCATTATATATCGTC
shSTING	GCATCAAGAATCGGGTTTATT	AATAAACCCGATTCTTGATGC
**sgRNA**		
sgPRMT3-1	GGTTCTGAAATAAGAGTCTT	
sgPRMT3-2	GGCTGATAGTGCAAATGTCA	
sgNC	GACCGGGGCGAGGAGCTGTTCACCG	

Supplementary Table 4. The primers used for CHIP assay in present study

Primer names	Forward (5'-3')	Reverse (5'-3')
PRMT3	TGGGCCAGGCCACATATTA	ACCCCACAGCACTGAACAAT
Prmt3	CCTGACTACACCTCCACCAA	CTTTGGTGGGCAGTTCCTCT
