# Supplementary material for: Targeting PRMT3 impairs methylation and oligomerization of HSP60 to boost anti-tumor immunity by activating cGAS/STING signaling
Source: Nat Commun. 2024 Sep 10;15:7930. doi: 10.1038/s41467-024-52170-3 (PMC11387718; doi:10.1038/s41467-024-52170-3)
Supplement: Supplementary file 3 — Description of Additional Supplementary Files [file 41467_2024_52170_MOESM3_ESM.pdf]

Description of Additional Supplementary Files

File Name: Supplementary Data 1
Description: Proteins detected by IP in HCC cell lysates followed mass spectrometry.

File Name: Supplementary Data 2
Description: Protein modifications detected by IP in HCC cell lysates followed mass spectrom etry.
